# Complementary therapies for bladder pain syndrome: a systematic review

**DOI:** 10.1007/s00192-015-2886-3

**Published:** 2015-12-07

**Authors:** Tina S. Verghese, Richael Ni Riordain, Rita Champaneria, Pallavi M. Latthe

**Affiliations:** University of Birmingham, Birmingham, UK; Newham University Hospital, Barts Health NHS Trust, Birmingham, UK; Birmingham Clinical Trials Unit, Birmingham University, Birmingham, UK; Birmingham Women’s NHS Foundation Trust, Birmingham, UK; School of Clinical and Experimental Medicine, College of Medical and Dental Sciences, The University of Birmingham, B15 2TT Birmingham, UK

**Keywords:** Alternative or complementary therapies, Myofascial physical therapies, Acupuncture, Bladder pain syndrome

## Abstract

**Introduction and hypothesis:**

Bladder pain syndrome is a difficult condition to treat. The purpose of this systematic review is to assess the effectiveness of various complementary therapies available for treatment.

**Methods:**

This review was conducted in adherence with Preferred Reporting Items for Systematic Reviews. Citations were retrieved using a comprehensive database search (from inception to July 2014: CINAHL, Cochrane, EMBASE, Medline and SIGEL and grey literature). Studies that fulfilled the inclusion criteria were selected. Eligibility consisted of women with bladder pain syndrome, an intervention of alternative/complementary therapies and an outcome of improvement of symptoms. Information regarding study characteristics and primary outcomes was collated. The Cochrane risk of bias scale was used to evaluate the quality of the studies included.

**Results:**

A total of 1,454 citations were identified, 11 studies fulfilled the inclusion criteria (4 randomised control trials [RCTs] and 7 prospective studies). The key interventions studied were acupuncture, relaxation therapy, physical therapy, hydrogen-rich therapy, diet and nitric oxide synthetase.

**Conclusion:**

Therapies with the potential for benefit in patients with bladder pain syndrome are dietary management, acupuncture and physical therapy. These findings were obtained from small studies and hence caution is advised. Robustly designed multicentre RCTs on these complementary therapies are needed to guide patients and clinicians.

## Introduction

The European Society for the Study of Interstitial Cystitis/Bladder Pain Syndrome in 2008 defined bladder pain syndrome (BPS) as pelvic pain, pressure or discomfort perceived to be related to the bladder, lasting for at least 6 months, and accompanied by at least one other urinary symptom [1]. Urinary symptoms include the persistent urge to void or frequency, in the absence of other identifiable causes. The International Urogynaecological Association (IUGA) and the International Continence Society (ICS) produced a joint report on terminologies by Haylen et al. in 2010, defining bladder pain as a “complaint of supra pubic or retro-pubic pressure, discomfort or pain, associated with the bladder, generally aggravated by bladder filling. The symptom may persist or alleviate after voiding.” [2]. An estimated 400,000 people in the UK suffer from BPS, the majority being women [3]. There is no definitive evidence to support an autoimmune, inflammatory, structural or infectious aetiology. Consequently, treating these patients is often challenging.

The interest in complementary and alternative therapies among patients is high and the number of effective treatments available for BPS are few [4]. Complementary and alternative therapies are an essential addition to the therapeutic armamentarium and include dietary modification, bladder training, acupuncture and stress reduction [5]. In 1995, complementary and alternative medicine (CAM) was defined by the National Institutes of Health (NIH) Center for Complementary and Alternative Medicine as, those treatments and healthcare modalities not widely taught or practised in medical schools or hospitals, and not usually reimbursed by medical insurance companies [6].

Our aim was to conduct a systematic review to evaluate the effectiveness of complementary therapies in the treatment of BPS.

## Materials and methods

This review was planned, conducted and reported in adherence with widely recommended methods [7]. No ethical approval was needed.

### Identification of studies

The following bibliographic databases were searched, from database inception to July 2014: CINAHL, Cochrane, EMBASE, Medline and SIGEL. Search strategies consisted of MeSH subheadings, text words and word variations for the concepts of BPS and alternative/complementary therapies. The basic search strategy was adapted to suit the database being searched. Librarians at the Royal College of Obstetricians and Gynaecologists (RCOG) performed the database searches. The search terms utilised by the RCOG: (Interstitial cystitis OR painful bladder syndrome OR bladder pain syndrome) AND Analgesia OR Pain relief OR Diet OR caffeine OR citrus OR Alcohol OR Smoking OR nicotine OR tobacco OR Physical therapy OR massage OR cupping OR exercise OR bladder training OR pelvic floor exercise OR Kegel exercise OR Stress management OR Psychology OR CBT OR talking OR counselling OR forums OR support groups OR acupuncture OR L-arginine. The search was restricted to “humans” and “females”. Bibliographies of relevant primary articles were also searched to identify any articles missed by the electronic searches. The conference proceedings and abstracts of the International Continence Society (2004–2014) and International Urogynecological Association meetings (2004–2014) were also searched. A comprehensive database was constructed using Endnote X7.2 for Windows, released 30 September 2014; Mac release date: 30 September 2014 [8] to store all references identified. No language restrictions were applied.

### Study selection and data extraction

Studies were selected following a two-step process. Firstly, the citations identified by the electronic bibliographic database searches were screened, based on their titles and abstracts. Full text papers of eligible abstracts were retrieved. Once full text papers had been located, we determined whether they fulfilled our predetermined inclusion criteria:

- Population: women with BPS or interstitial cystitis (IC).
- Intervention: complementary or alternative therapies
- Comparator: no treatment, another therapy
- Outcome: improvement in bladder symptoms
- Study designs: randomised controlled trial (RCT), cohort studies, case control studies, case series
- Exclusion criteria: pharmacological therapy, intravesical therapy, hydrodistention, tibial nerve stimulation

Two reviewers (TV, RNR) independently assessed the full text papers to determine if they met the above criteria. Any disagreements regarding the eligibility of a paper were solved through either consensus or arbitration by a third reviewer (PML). Data from the manuscripts included were extracted independently onto a pre-designed pro-forma. The literature search was thorough and without language restrictions. The grey literature was also searched and the data were extracted by two reviewers independently to reduce bias.

Data were collated on study characteristics, including methods of recruitment, patient characteristics, details of complementary therapy, outcomes and results. We contacted primary authors via email for any further information/clarification that was needed.

### Methodological quality

The methodological quality of all the papers fulfilling the inclusion criteria was assessed. Quality was defined as the confidence that the study design, conduct and analysis minimised bias in the estimation of effectiveness. Quality was assessed using existing checklists and scales [9, 10].

The methodological quality of the RCTs included was assessed using the Cochrane risk bias score [9]. The methodological quality of the non-RCT studies included was assessed using the Newcastle–Ottawa scale [10], where a non-randomised study was considered to be of high quality if it provided information on selection, comparability, exposure and outcome of the study participants. The quality checklist awards one star as a maximum for all items except comparability, where a maximum of two stars can be awarded. The scale was used to give a quantitative appraisal of overall quality of the non-randomised studies. The score ranged from 0 to 9, with a score of either 0 or 1 for each item.

### Data synthesis

We grouped studies according to their type of intervention and the comparison. The comparisons were with no intervention, with placebo or with another intervention. The outcome measures were based on the responses from questionnaires such as the Visual Analogue Score (VAS) [11], the Interstitial Cystitis Symptom Index (ICSI)/O’Sant Leary questionnaire [12], the Interstitial Cystitis Problem Index (IPSI) [12] and the maximum voided volume (MVV) on the frequency volume chart.

## Results

**Fig. 1:**
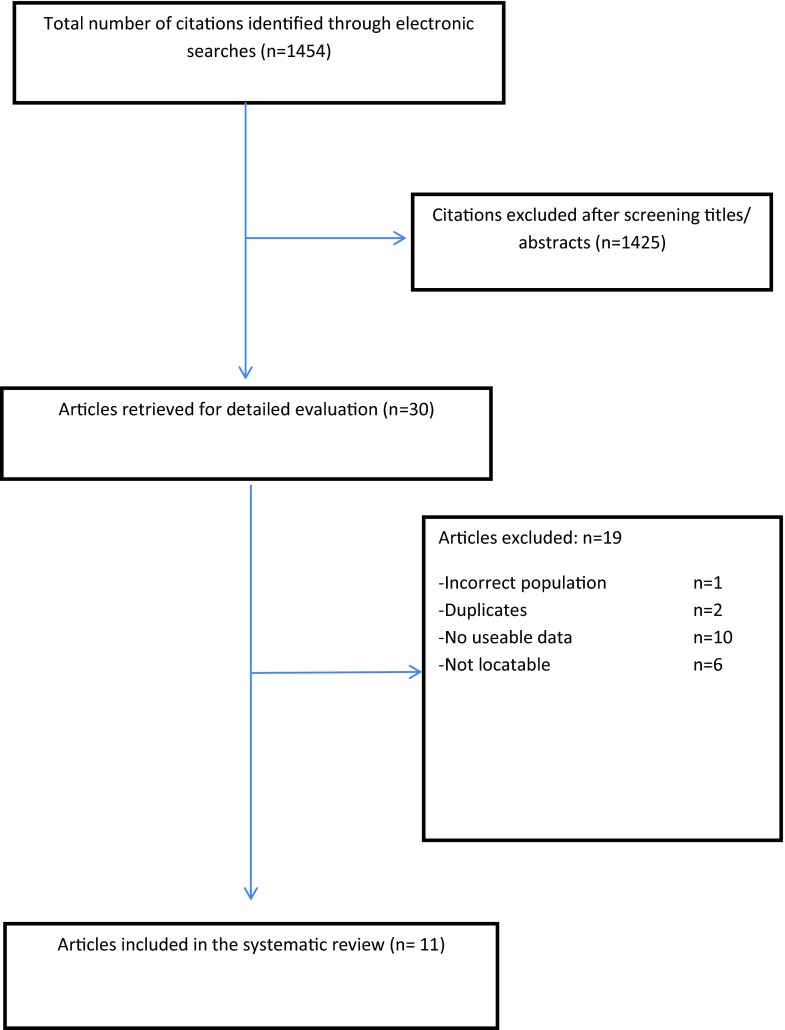
Flow process of the review

Out of the 1,454 citations identified by electronic literature searches, 11 studies that fulfilled the inclusion criteria were included in the systematic review (Fig. 1); 4 were randomized controlled trials (RCTs) and 7 were prospective studies. Table 1 provides a summary of the characteristics of the studies included. The mean age of the women ranged between 43 and 64 years. The criteria for the diagnosis of BPS varied in the studies included. Some studies diagnosed BPS clinically based on bladder pain scale, frequency and urgency. Other studies confirmed PBS based on the cystoscopic and hydrodistention findings. Because of the varied definitions utilised in last 5 years, we set the inclusion criteria for women with a diagnosis of BPS to be based on one of the following definitions: Hanash and Pool [13, 14], Messing and Stamey [15], the ICS [2] or NIH criteria [16], or the ESSIC definition (Table 1) [1]. Outcomes were documented as per the symptom-specific scores (VAS score, ICSI and ICPI).

**Table 1:** Characteristics of studies included in the systematic review on complementary therapies in painful bladder syndrome

Reference	Type of study	Population sample size	Age	Description of how BPS was diagnosed	Methods	Intervention	Control	Outcome
Korting et al. [26]	Double-blind, placebo controlled, randomised study	53/282 screened women with BPS were randomised	Median age in the L-arginine group 46 years and in the placebo group 52 years	Women with BPS were notified by an urologist or the Interstitial Cystitis Association to participate in the trial based on NIH criteria. No further information on how BPS was diagnosed in this group has been reported	Participants were given either L-arginine (1,500 mg) or placebo three times a day for 3 months. Outcomes were assessed at the end of 3 months. Follow-up was conducted before unblinding at 3.5 ± 2.0 months after completion of participation	L-arginine (*n* = 21/27); 6 withdrawals	Placebo (*n* = 25/26); 1 withdrawal	29 % of the L-arginine group and 8 % of the placebo group had clinically improved by the end of the trial. Likert scale of greater global improvement in the L-arginine group (48 %, 10 out of 21) compared with placebo (24 %, 6 out of 25) at 3 months (*p* = 0.05). Decrease in pain intensity (*p* = 0.04). Improvement in urgency (*p* = 0.06) and frequency of pain (*p* = 0.09)
Carrico et al. [21]	Prospective, randomised, controlled study	30 women with BPS enrolled and randomised to two equal groups (5 withdrawals in total)	Mean age = 44 years	BPS was diagnosed by cystoscopy and hydro-distension performed by a board-certified urologist	Guided imagery group listened to 25 minutes of guided imagery created for women with BPS, twice a day for 8 weeks. Control group rested for 25 minutes twice daily for 8 weeks. Participants were assessed at baseline and the end of 8 weeks with ICSI, ICPI VAS score and GRA	Guided imagery; *n* = 11/15	Control group; *n* = 14/15	45.5 % (5/11) of the treatment group compared with 14.3 % (2/14) of control group showed moderate to marked improvement in GRA. IC Self-efficacy scale – scores improved in both groups (not statistically significant). VAS pain score improved more in the treatment group (statistically significant *p* = 0.027). Urgency in voiding diaries = significantly declined in the treatment group (16 to 12 voids *p* = 0.02) whereas the control group showed no significant change (9.77–9.04 voids *p* = 0.684)
Matsumoto et al. [22]	Prospective, randomised, double-blind, placebo-controlled study	30 women with BPS were recruited	Mean age = 64 years	BPS diagnosis was based on cystoscopic findings during bladder hydrodistention	Participants were randomised in a ratio of 2:1 to have hydrogen-rich water or placebo. Each received 3 packs per day (1 pack = 200 ml) for 2 months. Symptoms were assessed at the end of 2 months based on ICSI, ICPI and VAS scores.	Hydrogen-rich water; *n* = 20	Placebo water; *n* = 10	Hydrogen-rich water did not show a significant difference compared with placebo water. Only 3 cases with hydrogen-rich water showed improvement in the VAS
FitzGerald et al. [23]	Single-blind, randomised, control study	81/ 585 screened women with BPS were randomised	Median age = 43 years	Clinical diagnosis of BPS and recorded ratings for bladder pain, frequency and urgency of at least 3 on a scale of 0 to 10 present for a minimum of 3 months and not more than 3 years	Each participant received ten 60-min sessions of intervention or control for 12 weeks. Participants were assessed using a 7-point global response assessment scale, ICSI and ICPI at 12 weeks, and followed up 3 months later	MPT(*n* = 38)	GTM (global therapeutic massage); *n* = 40	MPT: 59 % improvement (moderate to marked) in overall symptoms. GTM: 26 % overall improvement in symptoms
Ervan et al. [24]	Prospective, non-randomised study	37 women with BPS	Age range not mentioned	Women with BPS diagnosis based on clinical symptoms of urinary urgency, frequency and pain	Participants were treated with physical therapy. Outcome was assessed based on ICSI and ICPI (pre- and post-treatment scores recorded at the start of therapy and on completion of physical therapy at 3 months)	Participants given physical therapy; (*n* = 37)	–	ICSI score decreased from (median range) 12 to 6 (*p* < 0.001). ICPI score decreased from (median range) 10 to 7 (*p* < 0.001)
Shorter et al.[31]	Qualitative study	104 women with BPS	Mean age of the respondents was 54 years	Women with BPS based on clinical diagnosis	Participants completed a validated questionnaire designed to detect if food, beverages or supplement had an effect on bladder symptoms. A total of 175 comestibles were accounted for in the questionnaire. Women were asked to indicate whether each of the individual items worsened, improved or had no effect on symptoms. Each response was numerically scored on a scale of −2 to 2 and mean values were generated for each comestible item. Bladder symptoms were assessed based on ICSI scores	104/327 responded to the questionnaire	No control	90.2 % indicated that the consumption of certain foods or beverages caused symptom exacerbation. Participants who reported that specific foods worsened symptoms tended to have higher ICSI and ICPI scores. A total of 35 comestible items had a mean score of lower than 1.0, including caffeinated, carbonated and alcoholic, beverages, certain fruits and juices, artificial sweeteners and spicy foods
Honjo et al. [17]	Prospective cohort study	36 women, 11 with BPS	Ages ranged from 29 to 78 years	Women with BPS based on clinical diagnosis	Acupuncture performed using disposable stainless steel needles (0.3 mm, 60 mm in length, SEIRIN Kasei, Japan). Needles inserted into the bilateral BL33 points standardised by the WHO on the skin of the third posterior sacral foramina. Treatment repeated once a week for 4 weeks. Outcomes were assessed using VAS, bladder diary recording	Acupuncture (*n* = 30)	No control	24-h frequency significantly decreased from 15 to 9.8 times (*p* < 0.001) a day. VAS reduced significantly (*p* < 0.01)
Staack et al. [18]	Prospective cohort study	7 women with BPS were enrolled in the study	Mean age not documented	Clinical diagnosis of BPS and recording of bladder pain relieved on voiding, frequency and urgency recordings for at least 9 months.	Each participant received acupuncture (neuromodulation) for 3 months. Participants were assessed using pre- and post-treatment validated tools, such as the ICPI, ICSI, and pain was assessed using the VAS score	Acupuncture (*n* = 7)	No control	No significant change in the ICSI and ICPI. Improvements were observed in frequency (37.5 % *p* = 0.076), difficulty emptying (42.9 % *p* = 0.465) and genital pain (30 % *p* = 0.102). 3 months’ acupuncture showed modest improvement in overall urinary symptoms and painful bladder
Katayama et al. [19]	Prospective cohort study	8 women with BPS	Mean age = 62.9 ± 5 years	Women with BPS who failed to improve with medical treatment such as hydrodistention, intravesical instillation and pharmacological treatment	Each woman received acupuncture and moxibustion treatment. This was performed by applying moxa needles to BL32 and BL33 and performing electro-acupuncture on BL34 at 3 Hz for 20 min. Treatment given once every 2 weeks for 3 months. Outcomes assessed using ICSI, ICPI, VAS and maximum voided volume	Acupuncture + moxibustion (*n* = 8)	No control	3 responders (women with a reduction of VAS >2 and an increase in the maximum voided volume of >100 ml were considered to be responders). In these responders: VAS decreased 6 to 0 and 10 to 0; ICSI improved 10 to 0 and 11 to 3; IPSI improved 12 to 3 and 6 to 2. In responders, no recurrence was noted for 24 months
Ueda et al. [27]	Prospective cohort study	61 women with BPS	Mean age = 61.6	Women with BPS diagnosed based on symptoms recorded in 2-day voiding diaries and urine pH. Women with 8 or more micturitions per day and urine pH of less than 6.2. Participants fulfilled symptom-based diagnostic criteria of BPS	Participants were given citrates (a mixture of potassium citrate and sodium citrate) for 4 weeks to increase urine alkalinisation. Outcomes were assessed using King’s Health Questionnaire (KHQ), ICSI and ICPI	Participants who progressed to the treatment phase (*n* = 50)	–	Mean pH significantly increased from 5.6 to 6.0 *p* < 0.01, symptom improvement noted. Volume per void increased. ICPI and ICSI decreased significantly. Mean overall pain score decreased from 5.1 to 3.7
Lee et al. [25]	Prospective cohort study	56 women with BPS	Average age between 35 and 40 years	Women with BPS diagnosed using NIDDK-NIH criteria	The 56 women were divided into 3 groups (group 1 = 28, group 2 = 12 and group 3 = 16). They were to receive TVBF and TENS for the duration of 1, 2 and 3 months respectively. All women were given TVBF twice a day and TENS twice a week. Follow-up at 12 months, Outcomes assessed using ICSI, ICPI, VAS and GRA. Self-reported sexual activity were collected at baseline, and at the 3rd, 6th, 9th and 12th months	They were to receive TVBF and TENS Group 1: (*n* = 28) Group 2: (*n* = 12) Group 3: (*n* = 16)	–	ICSI, ICPI VAS and urgency score decreased significantly after TVBF + TENS in each group at the 3rd, 6th, 9th and 12th months compared with baseline (*p* = 0.05). GRA was 71 %, 70 %, 40 % at 12th month respectively. Statistically significant increase in self-reported sexual activity was noted at the 12th month compared with the 3rd month. Combination of TVBF and TENS for more than 2 months was not beneficial in the long term

*BPS* bladder pain syndrome, *ICSI* Interstitial Cystitis Symptom Index,* ICPI* Interstitial Cystitis Problem Index;* VAS* visual analogue scale,* NIDDK-NIH* National Institute of Diabetes and Digestive and Kidney Diseases–National Institutes of Health,* GRA* graph response assessment,* MPT* myofascial physical therapy,* GTM* global therapeutic massage,* TVBF* transvaginal biofeedback,* TENS* transcutaneous electric nerve stimulation

Tables 2 and 3 provide details of the methodological quality of the RCTs included. Table 4 summarises details of the quality of the non-randomised studies included.

**Table 2:**
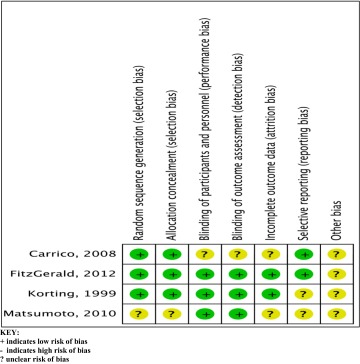
Risk of bias summary of the randomised control studies included

KEY:

+ indicates low risk of bias

- indicates high risk of bias

? unclear risk of bias

**Table 3:**
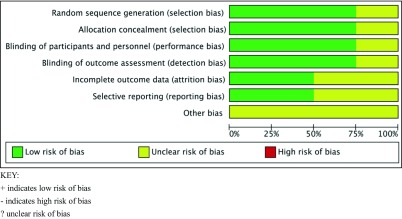
Risk of bias graph of the randomized control studies included

**Table 4:** Quality assessment of the non-randomised studies (Newcastle–Ottawa scale)

Study	Selection				Comparability		Outcome			Score
	Representativeness	Selection of non-exposed cohort	Ascertainment of exposure	Outcomes of interest	Controls	Additional factors	Assessment of outcome	Follow-up	Adequacy of follow-up	Out of 13
Ervan et al. [24]	*	x	*	*	*	x	* record linkage	*	x	6
Shorter et al.[31]	*	X	*	*	*	X	*	*	X	6
Honjo et al. [17]	*	x	*	*	*	x	*	x	x	5
Staack et al. [18]	*	x	*	*	*	x	*	*	*	7
Katayama et al. [19], Japan 2011	*	x	*	*	*	x	* record linkage	*	x	6
Ueda et al. [27]	*	x	*	*	*	x	* record linkage	*	x	6
Lee et al. [25]	*	x	*	*	X	x	*	X	x	4

*Indicates that a feature is present; x, that a feature is absent. But for comparability by design this checklist awards a maximum of two stars (**), one (*) or none if the feature is completely absent (x)

### Acupuncture and relaxation therapy

Honjo et al. investigated the clinical impact of sacral acupuncture on urinary sensory dysfunction on a cohort of 36 patients, 11 of whom had BPS (see Table 1). The treatment was repeated weekly for 4 weeks. At the end of treatment there was a significant decrease in the 24-h frequency and VAS for pain (*p* < 0.001) [17]. However, the results for the BPS and overactive bladder patients were presented together, preventing an assessment of symptoms in patients with BPS alone. Similarly, Staack et al. reported on a pilot study of seven IC patients in which 3 months of weekly acupuncture treatment with electric stimulation led to modest improvement in the urinary frequency, voiding difficulty and abdominal/genital pain [18]. Katayama et al. examined the effectiveness of acupuncture and moxibustion in 8 women with refractory BPS. 38 % of women showed improvement in symptoms after 3 months [19].

In a study conducted at the University of Tennessee, 20 patients with IC were hypnotised every 2 weeks for 2 months. Outcomes measured were a reduction in the analgesic requirements, increase in sleep time and quality of life (QoL). The majority of patients (95 %; 19 out of 20) were amenable to hypnotherapy and subsequently experienced pain reduction. Five patients reported a significant improvement (8–10 to 0–1 out of 10) on VAS and 85 % (*n* = 17 out of 20) were able to reduce their intake of analgesics by 25–50 % [20]. Carrico et al. conducted a pilot RCT in which more than 45 % of the treatment group responded to “guided imagery”, with a moderate or marked improvement in the global response rates (Table 1). In addition, the treatment group had a significant reduction in their ICSI (*p* = 0.006) and ICPI scores (*p* = 0.004) [21].

### Hydrogen-rich therapy

Matsumoto et al. conducted an RCT (30 women) of hydrogen-rich water versus placebo water for 2 months. The authors describe the process of the production of hydrogen-rich water. This was produced by passage through a reverse osmosis, an ion-exchange resin, and an ultrafiltration membrane. Hydrogen-rich pure water was then produced from dissolving hydrogen gas directly into pure water. To prevent the loss of hydrogen, the water is sealed in aluminium pouches and stored at room temperature. The study demonstrated no significant differences between treatment and placebo (Table 1) [22].

### Physical therapy

FitzGerald et al. conducted a multicentre RCT (*n* = 81 women) to determine the efficacy of myofascial physical therapy (MPT) compared with global therapeutic massage (GTM) [23]. The MPT group underwent connective tissue manipulation to all body wall tissues of the abdominal wall, back, buttocks and thighs that were clinically found to have connective tissue abnormalities to painful myofascial trigger points. Manipulation was applied bilaterally with the patient in the prone and then repeated in the supine position. This was performed until a texture change was noted. Manual trigger point release techniques were utilized to treat any noted trigger points or scars in the anterior or posterior lower quadrants. In the MPT arm, the therapists tailored the therapy to target patient needs.

Patients randomised to the GTM group received weekly massages consisting of full-body Western massage for 1 h. The therapist utilised techniques such as effleurage, petrissage, friction, tapotement, vibration and kneading.

At the end of 12 weeks, the global response rate was 26 % in the GTM group and 59 % in the MPT (*p* = 0.0012) group. Both groups reported improvement in secondary outcomes, including pain, urgency, frequency and QoL [23].

Similarly Ervan et al. conducted a non-randomised study on women with BPS. The physical therapy included connective tissue mobilisation over the trunk, thighs, buttocks and the release of trigger points, the resolution of adverse neural tension along the pudendal nerve, rehabilitation of the pelvic floor and hip girdle musculature. ICSI scores fell from median range of 12 before treatment to 6 post-therapy (*p* < 0.001). The ICPI scores decreased from 10 to 7 after therapy (*p* < 0.001) [24].

Women with BPS tend to have hypertonic pelvic floor muscle dysfunction. In these women, a combination of transvaginal biofeedback and transcutaneous electric nerve stimulation for more than 2 months has not been shown to have a long-term beneficial effect [25].

### L-arginine

Korting et al. undertook an RCT to investigate the efficacy of L-arginine, which is a substrate for nitric oxide, in which there were no differences between the groups according to intention-to-treat analysis (Table 1) [26].

Ueda et al. in their prospective study administered citrates to elevate the urine pH (Table 1). Urine alkalinisation was shown to be effective at improving symptoms of BPS [27].

Sacral acupuncture is a safe and promising therapeutic alternative, particularly in patients with PBS who have symptoms that do not respond to conventional treatments [17]. Acupuncture inhibits the transmission of pain and normalizes sensory processing within the peripheral and/or central nervous system. Patients with BPS display tension and tenderness of the pelvic floor musculature and connective tissue restrictions of muscle, fascia and subcutaneous tissue of the pelvic floor. Physical therapies, such as MPT, were found to be beneficial in women with symptoms of BS and associated pelvic floor tenderness [23].

## Discussion

To date there has been a paucity of studies addressing the effectiveness of complementary therapies in BPS. Results obtained from the small-scale studies we reviewed must be considered with caution because of the number of participants. Moreover, the definition of BPS in various studies is not uniform. Several unanswered questions regarding complementary therapies exist including, its applicability to all patients with BPS and the cost-effectiveness of treatment. The majority of studies reviewed undertook a limited period of follow-up prohibiting analysis of the long-term efficacy of the treatment. Studies evaluating the role of tibial nerve stimulation were excluded, as this has been explored in a recent systematic review [28].

The studies included did not discuss details of adverse effects of complementary therapies. However, in 2009 a group of CAM researchers conducted a workshop to discuss the challenges and safety issues of CAM. They found a low incidence of harmful/serious side effects from CAM [29]. Prospective observational studies have been conducted to evaluate the harmful effects of acupuncture; it has been found to be relatively safe. The data from 2.2 million treatment sessions found a risk of harmful events to be 1 in 76,000 patients [30]. The most common side effects noted with acupuncture were minor, e.g. bleeding or haematoma.

Shorter et al. utilised a validated questionnaire (IC/nutrition questionnaire) in a survey to detect whether food, beverages and supplement affect bladder symptoms in patients with BPS [31]. Analysis of the scoring of individual comestible items revealed that most bothersome consumables were caffeinated, carbonated and alcoholic beverages, citrus fruits and juices, artificial sweeteners and spicy foods. These results were obtained from survey rating and therefore further research and studies are required for confirmation of the results of the surveys conducted. Relief of symptoms was noted following ingestion of alkaline agents, e.g. calcium glycerophosphate (Prelief®) or sodium bicarbonate (baking soda). Interestingly, 75 % of respondents indicated that they had allergies, including seasonal allergies, allergies to medications, animals or foods. The survey conducted on female twins (*n* = 9,349) found that tea consumption was associated with an increased risk of BPS (OR 1.74, 95%CI 1.24–2.44), although coffee consumption was not (OR 1.1, 95%CI 0.84–1.45) [32]. Former and current smoking was associated with a higher risk of BPS (OR 1.5, 95%CI 1.18–1.89 and OR 1.49, 95%CI 1.16–1.92 respectively) [33].

In 2009, the Interstitial Cystitis Association (ICA) conducted an Internet-based survey of complementary therapies. A total of 2,101 responded to the survey; 1,982 confirmed an IC diagnosis. 84.2 % had tried complementary therapies and 55 % said that physicians had recommended complementary therapies [4]. Therapies perceived to be helpful included dietary management, myofascial physical therapy, temperature-related therapies, acupuncture, stress reduction and exercise. Some supplements, such as calcium glycerophosphate (Prelief), vitamin D, fish oil and probiotics, were also considered to be helpful. Calcium glycerophosphate appears to reduce BPS symptoms in a patient whose symptoms are exacerbated by particular foods [34]. Tettamanti et al. reported their findings from a population-based study demonstrating that tea and smoking were positively associated with BPS [33].

Many interstitial cystitis (IC) patients resort to non-conventional therapies after the failure of conventional measures. Unfortunately, little research on complementary and alternative therapies in the treatment of IC exists [5] and this is confirmed by our systematic review. Complementary and alternative medical therapy for interstitial cystitis (IC) is multimodal and individualized. Patients should be advised about the lack of robust evidence on complementary therapies and encouraged to participate in on-going studies on various therapies related to BPS [35].

In view of the dearth of robust evidence, there is a need for adequately powered RCTs to assess the effectiveness of complementary therapies in women with BPS. Studies could focus on therapies demonstrating potential with the limited evidence to date including: L-arginine, acupuncture, hypnotherapy and dietary modifications.
